# libFLASM: a software library for fixed-length approximate string matching

**DOI:** 10.1186/s12859-016-1320-2

**Published:** 2016-11-10

**Authors:** Lorraine A. K. Ayad, Solon P. Pissis, Ahmad Retha

**Affiliations:** Department of Informatics, King’s College London, The Strand, London, WC2R 2LS UK

**Keywords:** Approximate string matching, Fixed-length approximate string matching, Dynamic programming, Software library

## Abstract

**Background:**

Approximate string matching is the problem of finding all factors of a given text that are at a distance at most *k* from a given pattern. Fixed-length approximate string matching is the problem of finding all factors of a text of length *n* that are at a distance at most *k* from any factor of length *ℓ* of a pattern of length *m*. There exist bit-vector techniques to solve the fixed-length approximate string matching problem in time $\mathcal {O}(m\lceil \ell /w \rceil n)$ and space $\mathcal {O}(m\lceil \ell /w\rceil)$ under the edit and Hamming distance models, where *w* is the size of the computer word; as such these techniques are independent of the distance threshold *k* or the alphabet size. Fixed-length approximate string matching is a generalisation of approximate string matching and, hence, has numerous direct applications in computational molecular biology and elsewhere.

**Results:**

We present and make available libFLASM, a free open-source C++ software library for solving fixed-length approximate string matching under both the edit and the Hamming distance models. Moreover we describe how fixed-length approximate string matching is applied to solve real problems by incorporating libFLASM into established applications for multiple circular sequence alignment as well as single and structured motif extraction. Specifically, we describe how it can be used to improve the accuracy of multiple circular sequence alignment in terms of the inferred likelihood-based phylogenies; and we also describe how it is used to efficiently find motifs in molecular sequences representing regulatory or functional regions. The comparison of the performance of the library to other algorithms show how it is competitive, especially with increasing distance thresholds.

**Conclusions:**

Fixed-length approximate string matching is a generalisation of the classic approximate string matching problem. We present libFLASM, a free open-source C++ software library for solving fixed-length approximate string matching. The extensive experimental results presented here suggest that other applications could benefit from using libFLASM, and thus further maintenance and development of libFLASM is desirable.

## Background

### Computational problem

Fixed-length approximate string matching (FLASM) is a generalisation of the classic approximate string matching (ASM) problem. While numerous algorithms exist to tackle the latter [1], few algorithms exist to tackle the former. Given two strings, a pattern *x* and text *t*, and a positive integer *k*, the ASM problem consists of finding all factors of *t* that are at a distance at most *k* from *x* with respect to a distance model. With FLASM, the problem instead focuses on identifying all factors of *t* that are at a distance at most *k* from *any factor* of some fixed-length *ℓ*≤*m* of *x*. For example, given a text *t*=ATGGCAAGT, a pattern *x*=AAGATG, and a factor length *ℓ*=3, the factors of length *ℓ* in *x* are AAG,AGA,GAT,ATG. Of these factors, only the first and last find exact matches in *t*. Given a positive integer *k*<*ℓ*, as the distance threshold for a match, there could be more matches. If *k*=1, the factor AGA would match AGT with one error. This is the FLASM problem.

In molecular biology, errors occur at a steady rate in genetic codes with the average single-nucleotide substitution mutation rate in humans estimated to be 1.20×10^−8^ per nucleotide per generation [2]. Apart from single-nucleotide substitution, there are nucleotide deletion and insertion mutations, but these occur much less frequently [2]. Algorithms that operate on genetic sequences need to work with datasets containing substitutions, insertions, and deletions. Although specialised error models exist for molecular biology [3], the edit [4] and Hamming distance [5] models are often used as reasonable accuracy-speed trade-offs; these models have practical applications beyond the scope of biology.

The edit distance model uses substitutions, deletions, and insertions of letters to find the minimum number of operations to make two strings match. While each of these different operations can have a different edit cost, an edit cost of 1 is often used for all operations; and we simply call this cost the “edit distance”. The Hamming distance model is a simplified variant of edit distance which only allows substitutions and likewise, a substitution has a cost of 1.

The FLASM problem can be solved efficiently under the edit [6] or the Hamming [7] distance model; both algorithms make extensive use of bit-vector techniques to compute the solution. These techniques take advantage of low-level processor operations on computer words. Fast bit-vector operations can substitute regular operations, slashing the time complexity by a factor of *w*, the computer word size. Furthermore, bit-vectors are used to encode alignment information succinctly, thus improving the space complexity of the algorithms by the same factor. Specifically, the problem can be solved in time $\mathcal {O}(m\lceil \ell /w \rceil n)$ and space $\mathcal {O}(m\lceil \ell /w\rceil)$ under both distance models. FLASM is well-suited to solving numerous problems in computational molecular biology, including, for instance, multiple circular sequence alignment (MCSA) [8] and motif extraction [9–11]. FLASM is also very useful for solving problems outside the confines of computational biology, such as approximate circular string matching [12] as well as implementing the Chang and Marr index [13]. A brief description of these applications follows below.

### Biological motivation

Circular sequences are found in various places in the domain of biology [14, 15]. The bulk of bacterial DNA is stored in a circular chromosome but bacteria have additional genes stored in plasmids, circular loops of double-stranded DNA. Bacteria may harbour a variety of plasmids, giving them distinctive properties such as the ability to produce toxins or resist the effects of antibiotics [16]. Viral genomes also happen to be circular and the ability to identify regions of interest helps us in the discovery of druggable sites. Circular DNA is also present in specialised organelles, chloroplasts and mitochondria, of eukaryotic plant and animal cells. Mitochondrial DNA (mtDNA), can be found inside mitochondria [17] and is commonly used in phylogenetic reconstruction and research into ancestry and evolution. Additionally, some proteins are noted to share homologous domains but do not align because of swaposins, domains that are relocatable and have the property of circular permutability [18, 19]. Additionally, some proteins bind on their *N* and *C* termini in order to form a circular chain [15]. The wide presence of the circular structures in biology attests the importance of analysing circular sequences and finding algorithms suitable for its study [20].

Circular sequences have no point of reference by which they are sequenced or aligned to one another and treating them as linear sequences leads to poor alignments. By identifying the correct rotation for a pair of circular sequences, sequence alignment can be carried out to produce more reliable results. This is evident when analysing the linearised human (NC_001807) and chimpanzee (NC_001643) mtDNA sequences which start at different biological regions. Without refining the sequences, the pairwise sequence alignment of the mtDNA using EMBOSS Needle [21] gives a similarity score of 85.1 % with 1,195 gaps. Aligning different rotations of the same sequences yields a similarity of 91 % with only 77 gaps [8]. MCSA involves aligning three or more circular sequences simultaneously, which is a common task in computational molecular biology. As similar to the standard setting, this alignment can be used to find patterns within protein sequences and specifically, identify homology between new and existing groups of related sequences [22]. Just as importantly, it helps in identifying novel regions or mutations that give a species or breed its distinctive properties or highlights the cause of disease. A few tools exist to tackle the MCSA problem [8, 23, 24].

Motif extraction (ME), or motif discovery, involves detecting overrepresented DNA motifs as well as conserved DNA motifs in a set of orthologous DNA sequences. Such conserved motifs may serve as potential candidates for transcription factor binding sites for a regulatory protein [25]. The pattern, which is usually fairly short, 5 to 20 base-long, can be located in different genes or several times within the same gene. ME, however, may also be relevant for extracting longer regions within DNA sequences. A study in [26] shows that there exist 481 regions longer than 200 bases that are absolutely conserved in the genomes of the human, rat, and mouse. This fact suggests the possibility of the existence of long motifs in the presence of substitutions, insertions or deletions, underscoring the necessity of ME for larger lengths. Many tools exist to tackle the ME problem for single motifs [9, 27–29].

In addition to this simple form of single motifs, structured motifs are another special type of DNA motifs. A structured DNA motif is made up of two (or even more) smaller conserved sites with a spacer (gap) located between these sites. The spacer is found in the middle of the motif due to the transcription factors binding as a dimer. This means that the transcription factor is made up of two subunits, having two separate contact points with the DNA sequence. A non-conserved spacer of mostly fixed or slightly variable length separates these contact points. Such conserved structured motifs may serve as potential candidates for transcription factor binding sites for a composite regulatory protein [30]. A few tools exist to tackle the ME problem for structured motifs [11, 31, 32].

ME can also be used on immunoglobulins and T cell receptors, which aid in the immunity of humans and other vertebrates. Immunoglobulins, or antibodies, have the main function of eliminating foreign objects and pathogens such as bacteria, by attaching to them and neutralizing their effects on the body [33]. Obtaining the structural information of an antigen is essential for monoclonal antibody engineering, which has proven successful for the treatment of diseases such as Rheumatoid Arthritis [34]. These antibodies are created by introducing synthetic DNA into specific regions of the antibody, proving that ME aids with the discovery of common regions in multiple antigens.

It is well-known that ASM has numerous applications in computational molecular biology [1]. Average-case optimal algorithms for ASM [35] are based on a pre-processing step, namely, implementing the Chang and Marr index [13]. This step involves constructing an array *D*[0..*σ* ^*q*^−1] of integers, such that for every string *s* of length *q* over an alphabet of size *σ*, *s* is searched for in pattern *x*, and *D*[*s*] contains the smallest distance between *s* and any match found in *x*. Storing *D* requires space $\mathcal {O}(\sigma ^{q})$ and computing it naïvely takes time $\mathcal {O}(\sigma ^{q} q m)$ for the edit and Hamming distance models.

Approximate circular string matching (ACSM) is the problem of finding all factors of a given text that are at a distance *k* from any of the *m* rotations of a given pattern of length *m*. ACSM has various applications such as finding permutations in proteins [36], but it has other uses outside of biology and has been applied extensively in pattern recognition (see [37], for instance). Many average-case algorithms exist to tackle the ACSM problem efficiently for low error ratios *k*/*m* [12, 35, 38, 39].

### Our contributions

In this article, we present and make available libFLASM, a free open-source C++ software library for solving FLASM under both the edit and the Hamming distance models. For the Hamming distance model, we use our own implementation (a preliminary version appeared in [40]) of the MaxShiftM algorithm [7], and for the edit distance model we generalise the SeqAn [41] implementation of Myers’ bit-vector algorithm [6] for approximate string matching. Both of these implementations are based on dynamic-programming approaches accelerated by the use of bit-vector techniques to achieve their goals. Also, both implementations are able to handle arbitrary factor lengths; in particular, factor lengths greater than the computer word size. Moreover we make the following contributions by providing extensive experimental results to support our claims.

- **a)** We incorporate libFLASM into a state-of-the-art tool to improve the accuracy of MCSA in terms of the inferred likelihood-based phylogenies;
- **b)** We incorporate libFLASM into a state-of-the-art tool for ME of patterns longer than what was previously possible;
- **c)** We show how libFLASM can be used efficiently for implementing the Chang and Marr index;
- **d)** We show how libFLASM can be used efficiently for ACSM with high error ratios.

### Technical background

We provide some definitions following Crochemore et al. [42]. We define a *string* *x* of *length* *m* as an array *x*[0..*m*−1] where every *x*[*i*], 0≤*i*<*m* is a *letter* taken from an *alphabet* *Σ* of size |*Σ*|=*σ*. String *ε* denotes the *empty string* which has length 0. Given a string *y*, we call string *x* a *factor* of *y* if there exist two strings *u* and *v*, such that *y*=*uxv*. Consider the strings *x,y,u*, and *v*, such that *y*=*uxv*. We call *x* a *prefix* of *y* if *u*=*ε*; we call *x* a *suffix* of *y* if *v*=*ε*. When *x* is a factor of *y*, we say that *x* *occurs* in *y*. Each occurrence of *x* can be denoted by a position in *y*. Specifically, we say that *x* occurs at the *starting position* *i* in *y* when *y*[*i*..*i*+*m*−1]=*x*. It is often relevant to note the *ending position* *i*+*m*−1.

A circular string of length *m* may be informally defined as a standard linear string where the first-occurring and last-occurring letters are wrapped around and positioned next to each other. Considering this definition, the same circular string can be regarded as a set of *m* linear strings, which are all considered to be equivalent. Similarly, given a string *x* of length *m*, we denote by *x* ^*i*^=*x*[*i*..*m*−1]*x*[0..*i*−1], 0<*i*<*m*, the *i*th *rotation* of *x* and *x* ^0^=*x*. For instance, by looking at the string *x*=*x* ^0^=baababac, we obtain the following rotations of *x*: *x* ^1^=aababacb, *x* ^2^=ababacba, *x* ^3^=babacbaa, etc.

A *q-gram* is defined as any string of length *q* over alphabet *Σ*. Given an alphabet *Σ* of size *σ* and a value *q*, a *de-bruijn sequence* *B*(*q*,*σ*) is a circular string of length *σ* ^*q*^ such that every possible *q*-gram appears as a sequence of consecutive letters exactly once [43]. Several algorithms exist for this construction requiring time linear with respect to the length of the output sequence; for instance, see [44].

#### Definition 1.

Given a string *x* of length *m* and a string *y* of length *n*≥*m*, the edit distance, denoted by *δ*_*E*_(*x,y*), is defined as the minimum total cost of operations required to transform one string into the other. The allowed edit operations are as follows:

- *Insertion*: insert a letter in *y*, not present in *x*; (*ε*,*b*), *b*≠*ε*
- *Deletion*: delete a letter in *y*, present in *x*; (*a*,*ε*), *a*≠*ε*
- *Substitution*: replace a letter in *y* with a letter in *x*; (*a,b*), *a*≠*b*,*and* *a,b*≠*ε*.

Here we only consider edit distance with unit-cost operations. Given an integer *k*>0, if *δ*_*E*_(*x,y*)≤*k* we say that *x* and *y* have at most *k* *differences*.

#### Definition 2.

Given a string *x* of length *m* and a string *y* of length *m*, the Hamming distance, denoted by *δ*_*H*_(*x,y*), is defined as the minimum number of *substitution* operations required to transform one string into the other.

Given an integer *k*>0, if *δ*_*H*_(*x,y*)≤*k* we say that *x* and *y* have at most *k* *mismatches*.

We provide some further definitions following Carvalho et al. [31]. We define a *single motif* as a string of letters on an alphabet *Σ*. We are given an integer value *k* denoting a distance threshold (error threshold). We then say that a motif on *Σ* *k*-occurs in another string *s* on *Σ*, if there is a distance (edit or Hamming, for instance) of at most *k* between the motif and a factor of *s*. A set *s*_1_,…,*s*_*N*_ of strings on *Σ*, where *N*≥2, the quorum 1≤*q*≤*N*, the error threshold *k*, and the length *ℓ* for the motifs are taken as input for the *single motif extraction* problem. Specifically, it involves identifying all motifs of length *ℓ*, with each motif *k*-occurring in at least *q* input strings. In this case, such single motifs are called *valid*.

We define a *structured motif* as a pair of the form (*m,d*): *m*=(*m*_*i*_)1≤*i*≤*β* is a *β*-tuple of single motifs; and $d = (d_{\min _{i}}, d_{\max _{i}})_{1 \leq i < \beta }$ is a *β*−1-tuple of pairs, which correspond to *β*−1 intervals of distance between the *β* single motifs. We denote a structured motif by

$$m_{1}[d_{\min_{1}}, d_{\max_{1}}]m_{2} \ldots m_{\beta - 1}[d_{\min_{\beta - 1}}, d_{\max_{\beta - 1}}]m_{\beta}. $$

The elements *m*_1_,*m*_2_,…,*m*_*β*_ of a structured motif are called *boxes*. The length of box *m*_*i*_ is denoted by *ℓ*_*i*_. We are given a *β*-tuple (*k*_*i*_)_1≤*i*≤*β*_ of error thresholds. We then say that a structured motif (*m,d*) has a (*k*_*i*_)_1≤*i*≤*β*_-occurrence in another string *s* on *Σ*, if there is a *k*_*i*_-occurrence $m^{\prime }_{i}$ of *m*_*i*_, for all 1≤*i*≤*β*, such that:

1. $m^{\prime }_{1}, \ldots, m'_{\beta }$ occur in *s* and
2. the distance between the ending position of $m^{\prime }_{i}$ and the starting position of $m^{\prime }_{i+1}$ in *s* is in interval $[d_{\min _{i}}, d_{\max _{i}}]$, for all 1≤*i*<*β*.

A set *s*_1_,…,*s*_*N*_ of strings on *Σ*, where *N*≥2, the quorum 1≤*q*≤*N*, *β* lengths (*ℓ*_*i*_)_1≤*i*≤*β*_, *β* error thresholds (*k*_*i*_)_1≤*i*≤*β*_, and *β*−1 intervals $(d_{\min _{i}},d_{\max _{i}})_{1 \leq i < \beta }$ of distance are taken as input for the *structured motif extraction* problem. Specifically, it involves identifying all structured motifs that have a (*k*_*i*_)_1≤*i*≤*β*_-occurrence in at least *q* input strings. In this case, such structured motifs are called *valid*. A problem instance is denoted by

$$\begin{aligned} &<(\ell_{1},k_{1})[d_{\min_{1}},d_{\max_{1}}](\ell_{2},k_{2}) \ldots \\&\quad\!\!\!\!(\ell_{\beta-1},k_{\beta-1})[d_{\min_{\beta-1}},d_{\max_{\beta-1}}](\ell_{\beta},k_{\beta}),q>. \end{aligned} $$

We are now in a position to define the FLASM problem.

#### Definition 3.

Given a pattern *x* of length *m*, a text *t* of length *n*≥*m*, an integer *ℓ*≤*m*, and an integer *k*<*ℓ*, the FLASM problem under the Hamming distance model finds all factors *u* of *t* such that *δ*_*H*_(*u,v*)≤*k*, where *v* is any factor of length *ℓ* of *x*.

We extend the above definition to the edit distance model.

#### Definition 4.

Given a pattern *x* of length *m*, a text *t* of length *n*≥*m*, an integer *ℓ*≤*m*, and an integer *k*<*ℓ*, the FLASM problem under the edit distance model finds all factors *u* of *t* such that *δ*_*E*_(*u,v*)≤*k*, where *v* is any factor of length *ℓ* of *x*.

#### Theorem 1 ([7]).

The FLASM problem under the Hamming distance model can be solved in time $\mathcal {O}(m\lceil \frac {\ell }{w}\rceil n)$ and space $\mathcal {O}(m\lceil \frac {\ell }{w}\rceil)$, where *w* is the size of the computer word.

Given a pattern *x* of length *m*, a text *t* of length *n*≥*m*, and an integer *k*<*m*, Myers bit-vector algorithm solves the ASM problem under the edit distance model in time $\mathcal {O}(n\lceil \frac {m}{w}\rceil)$ and space $\mathcal {O}(\lceil \frac {m}{w}\rceil)$ [6]. Applying this algorithm for all $\mathcal {O}(m)$ factors of length *ℓ* of *x* separately solves the FLASM problem under the edit distance model.

#### Theorem 2.

The FLASM problem under the edit distance model can be solved in time $\mathcal {O}(m\lceil \frac {\ell }{w}\rceil n)$ and space $\mathcal {O}(m\lceil \frac {\ell }{w}\rceil)$, where *w* is the size of the computer word.

### Applications

In this section we explain how Theorems 1 and 2 can be applied to solve problems in computational molecular biology and elsewhere.

#### Application I: multiple circular sequence alignment

MCSA is a generalisation of the multiple sequence alignment (MSA) problem. Informally, in MSA, the goal is to compare and visualise a set of *N* input sequences *s*_1_,…,*s*_*N*_ such that comparing their bases with each other reduces the total cost of this alignment through a minimal application of substitutions, insertions, and deletions. As obtaining an optimal MSA is computationally expensive (using dynamic-programming techniques) [45], an alternative approach is to use heuristic techniques [22, 46]. These techniques make an implicit assumption: the left- and right-most position of each sequence is relevant; however this does not necessarily apply to circular sequences. Circular genomes are split and sequenced at a possibly random position. So when it comes to comparing them, if they do not start with the same biological region, it is quite possible to obtain very sub-optimal MSAs. To resolve this and reduce the total cost of the produced MSA, the sequences can be refined to different rotations before going through the MSA algorithm. Theorems 1 and 2 can be applied to find most similar factors among pairs of sequences (*s*_*i*_,*s*_*j*_), which can then determine suitable rotations for all input sequences via agglomerative hierarchical clustering (see [8], for details).

#### Application II: motif extraction

For single ME, a factor of length *ℓ* can be considered as a single motif, extracted once or several times from a subset of *N* input sequences *s*_1_,…,*s*_*N*_. The extraction of motifs under the Hamming and edit distance models can be performed by applying Theorems 1 and 2, respectively, for all pairs of input sequences. Specifically, it suffices to perform all pairwise sequence comparisons (*s*_*i*_,*s*_*j*_) and store the number of occurrences for each factor of length *ℓ* of every sequence. We can thus determine all single motifs of length *ℓ*, such that each motif *k*-occurs in at least *q* of the input sequences. The assumption of this approach is that all reported motifs are strictly valid: they occur at least once in some sequence with no error (see [9], for details).

Structured ME is a generalisation of single ME. A structured motif is made up of single motifs with distance intervals of varying size between them. Theorems 1 and 2 can be applied to find occurrences of individual motifs *m*_1_,*m*_2_,…,*m*_*β*_, where *β* is the number of single motifs within a structured motif; and then merge these occurrences efficiently to form structured motifs. The same validity assumption holds for structured motifs (see [11], for details).

#### Application III: Chang and Marr index

The Chang and Marr index aims to find the minimal distance between every *q*-gram over *Σ* and any factor of pattern *x*. We propose here the following implementation. Given a pattern *x* of length *m* consisting of *σ* distinct letters and a value for *q*, the de-bruijn sequence *B*(*q*,*σ*) is constructed using the algorithm of [44]. We also initialise an integer array *D*: a numerical representation of the *σ* ^*q*^ *q*-grams permits constant time access to *D*. The minimal distance for each *q*-gram represented in *D* can be computed using the Hamming or edit distance between a factor of length *q* from the de-bruijn sequence and any factor of *x*. A linearised version of the de-bruijn sequence and pattern *x* can be used as the input pattern and text, respectively, for FLASM; we also need to set *ℓ*:=*q*. Hence the computation can be done in time $\mathcal {O}(\sigma ^{q} \lceil q / w \rceil m)$, instead of $\mathcal {O}(\sigma ^{q} q m)$, by applying Theorems 1 and 2.

#### Application IV: approximate circular string matching

ACSM is the problem of finding all factors of a text *t* that are at a distance *k* from any of the rotations of a pattern *x*. We can consider all rotations of *x* of length *m* by creating string *x* ^′^=*x*[0..*m*−1]*x*[0..*m*−2] and sliding a window of length *m* across *x* ^′^. Consider the pattern *x*=AAGATG; we obtain rotations: AAGATG,AGATGA,GATGAA,ATGAAG,TGAAGA,GAAGAT. The ACSM problem consists in searching for these rotations in *t*. Theorems 1 and 2 can be directly applied by using *x* ^′^ as the input pattern and setting *ℓ*:=*m*. This means all factors of length *ℓ*=*m* of *x* ^′^, hence all rotations of *x*, will be used to search in *t*.

## Implementation

libFLASM was implemented and packaged as a dynamic library in the C++ programming language and was compiled with gcc v.4.7.3 using optimisation flags -O3, -funroll-loops, and -msse4.2. The implementation is distributed under the GNU General Public License (GPL), and it is available freely at repository https://github.com/webmasterar/libFLASM.

libFLASM exposes two functions: one to solve FLASM under the edit distance model; and one for the Hamming distance model. Both functions require the following parameters to operate:

- *t* The text to search
- *n* The length of the text
- *x* The pattern text
- *m* The length of the pattern
- *ℓ* The length of the factor
- *k* The maximum distance allowed between a factor of *x* and a factor of *t*
- *r* A flag to indicate if all or the best matches should be returned

The functions return a set of tuples in the form <*j,i,e*>, where:

- *j* is the ending position of the match in *t*
- *i* is the ending position of the match in *x*
- *e* is the distance of the match

The edit distance function was trivially implemented using SeqAn [41], a free open-source C++ library of algorithms for sequence analysis. Specifically, we used the implementation of Myers’ bit-vector algorithm [6] to perform approximate string matching under the edit distance model. We adapted it to search for all factors of *x* of length *ℓ* in *t* in order to solve the FLASM problem. For the Hamming distance function, we used our own implementation [40] of the MaxShiftM algorithm [7], which solves the FLASM problem under the Hamming distance model. Notice that both functions provided by the library work for an arbitrary length *ℓ* using multiple computer words, thus delivering the $\mathcal {O}(m \lceil \frac {\ell }{w}\rceil n)$-time and $\mathcal {O}(m\lceil \frac {\ell }{w}\rceil)$-space complexity of the proposed algorithms.

libFLASM may easily be incorporated into any computational pipeline; some examples are given below.

### Incorporation of libFLASM into BEAR

libFLASM was incorporated into BEAR (BEst Aligned Rotations) [8], a state-of-the-art tool for improving MCSA. BEAR uses the library to find most similar factors, under a pre-specified distance model, between pairs of the input sequences, which can then determine suitable rotations for all input sequences. The output of BEAR can then be used as input for any MSA program.

BEAR takes a MultiFASTA file containing a set of *N* sequences *s*_1_,*s*_2_,…,*s*_*N*_ as input, performs all pairwise sequence comparisons, and stores the results in a matrix *M* of size *N*×*N*. Each cell *M*[*i,j*] stores information about the rotation and distance of every pairwise sequence comparison (*s*_*i*_,*s*_*j*_). This information is then used as input for standard agglomerative hierarchical clustering [47] in order to group closely-related sequences together and apply the rotations to output a refined dataset of the *N* sequences. This refined dataset can in turn be passed to an MSA program such as MUSCLE [46] or Clustal *Ω* [48] to produce the final MSA.

BEAR provides a selection of algorithms to do the pairwise sequence comparison. One of these approaches is based on FLASM. This was previously restricted to using only factors of length *ℓ*≤*w*, but with the incorporation of libFLASM, it is now possible to use arbitrary values for *ℓ* under the edit or the Hamming distance model.

### Incorporation of libFLASM into MoTeX-II

libFLASM was incorporated into MoTeX-II [11] (the successor of MoTeX [9]), a state-of-the-art tool to identify single and structured motifs. MoTeX-II uses the library to find occurrences, under a pre-specified distance model, of each factor of length *ℓ* of every sequence in the input sequences.

MoTeX-II takes a MultiFASTA file containing a set of *N* sequences *s*_1_,*s*_2_,…,*s*_*N*_ as input. For single ME, it performs all pairwise sequence comparisons and stores the number of occurrences for each factor of length *ℓ* of every sequence. Hence it can determine all valid motifs of length *ℓ*. This was previously restricted to finding motifs of length *ℓ*≤*w*, but with the incorporation of libFLASM, it is now possible to find motifs of any length *ℓ*. A similar approach is followed for structured ME.

### Using libFLASM to implement the Chang and Marr index

The libFLASM library repository contains an example showing the use of libFLASM to implement the Chang and Marr index, that is represented by an integer array *D* of size *σ* ^*q*^. Given a pattern *x* and a value *q*, the index can be implemented under the Hamming or edit distance model. The minimal distance between each *q*-gram in the linearised version of the de-bruijn sequence and any factor of *x* is computed using libFLASM by setting the factor length to *ℓ*:=*q*. A list of tuples of the form <*i,e*> can thus be created, where *i* is the ending position of a *q*-gram *s* in the de-bruijn sequence and *e* is the minimal distance found between *s* and any factor in *x*. By using a numerical representation of *s*, we can easily construct array *D*.

### Using libFLASM for performing approximate circular string matching

The libFLASM library repository comes with another example program that can be used to perform FLASM under a pre-specified distance model. There was no need to modify this program to make it suitable for ACSM. All that is needed is to double-up the pattern *x* of length *m*, by concatenating it with itself to create string *x* ^′^=*x*[0..*m*−1]*x*[0..*m*−2], and then set the factor length to *ℓ*:=*m*.

## Results and discussion

The experiments were run on a 64-bit GNU/Linux operating system on a machine with a quad-core 64-bit Intel 2.8GHz Core-I7 processor with 8GB of RAM.

### Experiment I: performance

**Fig. 1:**
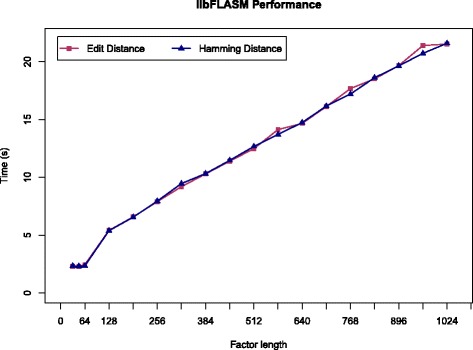
Experiment I. Elapsed time in seconds of libFLASM under edit and Hamming distance models for *n*=*m*=10,000 and increasing factor length *ℓ*

We first evaluated the performance of libFLASM by creating two random 10,000 base-long DNA sequences for the text *t* and the pattern *x*. Different factor lengths were used in the range 32,…,1,024. The distance threshold *k* was set to $\frac {1}{2}\ell $, while the length *n* of the text, the length *m* of the pattern, and the word size *w*=64 remained constant. The results shown in Fig. 1 confirm the theoretical findings.

The results clearly show the linear growth of the time required to complete the computation with respect to the factor length *ℓ* in accordance with the time complexity of $\mathcal {O}(m\lceil \frac {\ell }{w}\rceil n)$. Note also that the first three points, which use factors of length 32, 48, and 64 are represented on the graph as a straight horizontal line, indicating that there is no increase in the time required to compute them. This is trivially explained by the $\lceil \frac {\ell }{w}\rceil $ factor in the time complexity.

### Experiment II: approximate circular string matching

We then evaluated the performance of libFLASM against state-of-the-art algorithms for solving the ACSM problem. Two fast average-case algorithms, both of which support the edit and Hamming distance models, were identified from the literature: CMFN [35] and ACB [38]. The corresponding implementation of the algorithms were obtained via communication with the authors.

ACB is an algorithm designed with the purpose of solving the ACSM problem. CMFN is an algorithm designed to solve the general problem of multiple approximate string searching—that is, searching for a set of *N* patterns *x*_1_,*x*_2_,…,*x*_*N*_ simultaneously in the text. We thus adapted CMFN to search for all rotations of *x* in order to compare the speed of the three programs. This was done by considering all rotations of *x* as the set of input patterns. Unfortunately, the implementation of ACB is restricted to searching patterns of length less than or equal to the computer word size (*m*≤*w*).

We ran the three programs with the following settings. ACB was run with the option -k to set the distance threshold and the list of patterns were read in on the console from a file. CMFN was run with the options: -D -B -Sb -t 6 -k *k*. This uses a *q*-gram length of 6, which is noted for its suitability in [35]. Hamming distance was enabled using the option -s. To test libFLASM, we used the example application that is packaged with the library. The distance model, the factor length *ℓ*, and the distance threshold *k* options were set accordingly.

**Fig. 2:**
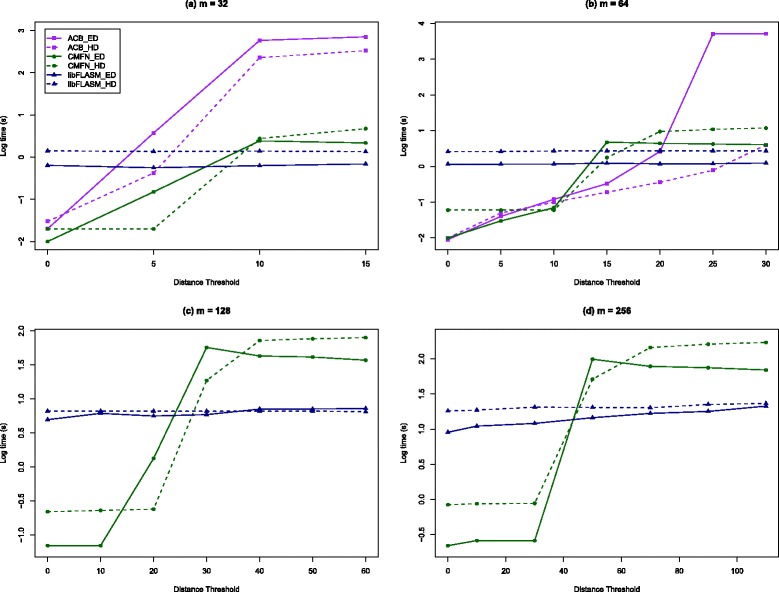
Experiment II. Elapsed-time comparison in log10 seconds of different programs for ACSM under edit and Hamming distance models for *m*=32,64,128,256, *n*=1,000,000, and increasing distance threshold *k*

A one million base-long DNA sequence was randomly generated as the text *t* to be searched. Patterns of length *m*=32,64,128,256 were randomly picked from *t*. In the case of libFLASM, each pattern was concatenated with itself and the factor length was set to *ℓ*:=*m*. With ACB, only 32 and 64 base-long patterns were tested because of the limitations of its implementation. Both the Hamming and edit distance versions of the programs were tested. The other parameter considered was the performance of the programs with regards to the distance threshold *k*. A series of values for *k* from the range ${0,\ldots,\frac {1}{2}\ell }$ was used. The results are shown in Fig. 2.

The results of this experiment show that ACB and CMFN are fast for small values of *k*, but once *k* is increased we find that libFLASM becomes competitive and goes on to perform significantly better. This is explained by the fact that the time complexity of ACB and CMFN depends on *k*, while libFLASM is independent of *k*. This is clearly captured by the graphs in Fig. 2. Apart from its speed and robustness, another benefit of using libFLASM, not shown here, is alphabet independence.

### Experiment III: multiple circular sequence alignment

Nine synthetic datasets were generated using INDELible [49] in order to test the accuracy of BEAR with libFLASM for improving MCSA. INDELible is a program which generates DNA or protein sequences with substitutions, insertions, and deletions at rates defined by the user. For input datasets we used files containing 12, 25, or 50 DNA sequences (number denoted by *α*) of length approximately 2,500 (length denoted by *γ*). Three unique substitution rates (denoted by *θ*) were applied, per dataset, using the substitution model JC69 [50], at 5 %, 20 %, and 35 %. The insertion and deletion rates were set, respectively, to 4 % and 6 % (denoted by *κ* and *ω*), relative to a substitution rate of 1. We will call these datasets the *Original* datasets.

We then proceeded to randomly rotate each of the sequences in the datasets to create a new set of files. We call these the *Random* datasets. The goal of this experiment was to use BEAR with libFLASM under the edit distance model to refine the random rotation of each of the sequences in the *Random* datasets. The refined datasets we would obtain after rotating the sequences are called the *Restored* datasets. We ran BEAR using the FLASM method for pairwise sequence comparisons under the edit distance model. We used two combinations of factor length *ℓ* and distance threshold *k* to run the experiments: *ℓ*=40, *k*=10; and *ℓ*=100, *k*=45.

We then used MUSCLE [46], a fast and accurate MSA program, to produce the alignments in PHYLIP format for each *Original* dataset. The alignments were then passed to RAxML [51], a program for inferring a phylogenetic tree, under the maximum likelihood criterion, for a given alignment. RAxML was used again to compare the trees against each other via calculating the pairwise Robinson and Foulds (RF) distance [52]. In particular we calculated the RF distance between the *Original* trees and the *Random* trees, as well as the distance between the *Original* trees and the *Restored* ones, to measure how well the programs had performed in refining the sequences in each of the datasets.

The results in Table 1 show the RF distances between the *Original* datasets and the *Random* datasets, shown in Column 1. The distance between the *Original* datasets and the *Restored* ones using Cyclope [23], another tool for MCSA, is shown in Column 2, and the distance between the *Original* datasets and the *Restored* ones using BEAR is shown in Columns 3 and 4. BEAR was run under the edit distance model with *ℓ*=40 and *k*=10, shown in Column 3 and again with *ℓ*=100 and *k*=45, shown in Column 4. Table 1 shows that BEAR produces very good results: an RF distance of 0 was obtained for all datasets when using *ℓ*=100. Table 1 shows the necessity of using a factor length larger than the word size, as it produces more accurate results than when using *ℓ*=40. This is expected as longer factors are more likely to provide information about reliable rotations than shorter factors, which could potentially have multiple occurrences with at most *k* differences. Elapsed-time comparisons are shown in Table 2. The results show that BEAR performs significantly faster than Cyclope. Ultimately, these results demonstrate that libFLASM can be applied effectively and efficiently in BEAR for improving MCSA.

**Table 1:** RF distance between the *Original* and *Random* datasets as well as the RF distance between the *Original* and *Restored* datasets using Cyclope and BEAR

Dataset <*α*,*γ*,*θ*,*κ*,*ω*>	*Random*	Cyclope	BEAR	BEAR
			*ℓ*=40	*ℓ*=100
<12,2500,0.05,0.06,0.04>	0.000	0.000	0.000	0.000
<12,2500,0.20,0.06,0.04>	0.000	0.000	0.000	0.000
<12,2500,0.35,0.06,0.04>	0.000	0.000	0.000	0.000
<25,2500,0.05,0.06,0.04>	0.000	0.000	0.000	0.000
<25,2500,0.20,0.06,0.04>	0.000	0.000	0.000	0.000
<25,2500,0.35,0.06,0.04>	0.045	0.045	0.000	0.000
<50,2500,0.05,0.06,0.04>	0.085	0.000	0.021	0.000
<50,2500,0.20,0.06,0.04>	0.043	0.000	0.000	0.000
<50,2500,0.35,0.06,0.04>	0.043	0.000	0.021	0.000

**Table 2:** Elapsed-time comparison in seconds of Cyclope and BEAR

Dataset <*α*,*γ*,*θ*,*κ*,*ω*>	Cyclope	BEAR *ℓ*=40	BEAR *ℓ* = 100
<12,2500,0.05,0.06,0.04>	79.09	15.92	46.53
<12,2500,0.20,0.06,0.04>	77.47	15.06	44.52
<12,2500,0.35,0.06,0.04>	76.76	14.85	45.44
<25,2500,0.05,0.06,0.04>	332.69	69.81	203.78
<25,2500,0.20,0.06,0.04>	342.94	69.28	208.85
<25,2500,0.35,0.06,0.04>	344.50	71.14	208.82
<50,2500,0.05,0.06,0.04>	1,317.81	293.45	851.07
<50,2500,0.20,0.06,0.04>	1,303.51	300.37	837.66
<50,2500,0.35,0.06,0.04>	1,359.90	286.88	854.83

### Experiment IV: motif extraction

We carried out experiments on real data retrieved from the Kyoto Encyclopedia of Genes and Genomes (KEGG) Database [53]. Three sets of data were obtained made up of: RNA polymerase proteins; viruses; and hypothetical proteins. Single ME was carried out on these datasets. All datasets contain 11 sequences: the first dataset is made up of RNA Polymerase sequences, with all sequences containing 4 distinct matching motifs of varying length; the second dataset is made up of virus sequences, all containing 4 matching motifs; the third dataset is made up of hypothetical proteins, containing 2 distinct matching motifs.

Table 3 shows the results obtained when parameters of the form <*ℓ*,*k*> were used to extract the single motifs from the datasets. The quorum shows the percentage of sequences which contained the listed motif. A quorum of 100 shows that for each set of input sequences, all sequences contained the same single motif, as expected. Table 3 shows that MoTeX-II was able to identify all motifs of various lengths when used with libFLASM. These real datasets show the necessity of using a factor length larger than the word size for ME.

**Table 3:** Single motif extraction from real datasets

Dataset	Parameters	Motif	Quorum (%)
RNA	<350,110>	RNA polymerase	100
		Rpb2, domain 6	
Polymerase	<60,28>	RNA polymerase	100
		Rpb2, domain 4	
	<40,12>	RNA polymerase	100
		Rpb2, domain 5	
	<90,30>	RNA polymerase	100
		Rpb2, domain 7	
Viruses	<350,150>	Viral methyltransferase	100
	<130,50>	Cucumber mosaic	100
		virus 1a protein	
	<70,48>	Cucumber mosaic virus 1a	100
		protein C terminal	
	<250,130>	Viral (Superfamily 1) RNA	100
		helicase	
Hypothetical	<130,45>	Type III restriction enzyme,	100
		res subunit	
Proteins	<60,30>	Helicase conserved	100
		C-terminal domain	

Synthetic data was also used to extract structured motifs from sequences. We generated 50 random 1,000 base-long DNA sequences. Structured motifs were implanted in half of the DNA sequences. Table 4 shows that the incorporation of libFLASM into MoTeX-II has allowed for structured motifs with lengths *ℓ*_1_,…,*ℓ*_*β*_>*w* to be extracted. The parameters are in the form $<(\ell _{1},k_{1})[d_{\min _{1}},d_{\max _{1}}](\ell _{2},k_{2})[d_{\min _{2}},d_{\max _{2}}](\ell _{3},k_{3})>$ where $[d_{\min _{i}},d_{\max _{i}}]$ represents the range of the distance interval allowed between each motif box. In each test, 25 structured motifs were implanted into the sequences and MoTeX-II was able to identify all of these structured motifs in all cases. The statistical significance test conducted using MoTeX-II or other tools is beyond the scope of this article.

**Table 4:** Structured motif extraction from synthetic datasets

Parameters	Implanted	Implanted
	structured	structured
	motifs	motifs
		extracted
<(80,15)[5,15](60,10)[5,20](230,20)>	25	25
<(100,15)[5,15](80,10)[5,20](250,20)>	25	25
<(120,15)[5,15](100,10)[5,20](270,20)>	25	25
<(140,15)[5,15](120,10)[5,20](290,20)>	25	25
<(160,15)[5,15](140,10)[5,20](310,20)>	25	25
<(180,15)[5,15](160,10)[5,20](330,20)>	25	25
<(200,15)[5,15](180,10)[5,20](350,20)>	25	25

### Experiment V: Chang and Marr index

We carried out experiments on synthetic data to test the implementation of the Chang and Marr index under the Hamming and edit distance models when using libFLASM in comparison to a naïve implementation. Table 5 shows the elapsed time to compute the Chang and Marr index for *q*-grams of lengths 5 to 10 under the Hamming and edit distance model, using a pattern of length 32. Table 6 shows likewise for a pattern of length 64. Both patterns were generated randomly (uniform distribution) over the DNA alphabet.

**Table 5:** Elapsed-time comparison in seconds for implementing the Chang and Marr index using a pattern of length 32

	Edit distance		Hamming distance	
*q*-gram length	Naïve (s)	libFLASM (s)	Naïve (s)	libFLASM (s)
5	0.01	0.00	0.01	0.00
6	0.08	0.02	0.08	0.01
7	0.67	0.9	0.55	0.05
8	6.20	0.50	4.81	0.25
9	34.00	2.74	23.99	1.43
10	145.56	11.76	96.71	6.24

**Table 6:** Elapsed-time comparison in seconds for implementing the Chang and Marr index using a pattern of length 64

	Edit distance		Hamming distance	
*q*-gram length	Naïve (s)	libFLASM (s)	Naïve (s)	libFLASM (s)
5	0.04	0.01	0.04	0.00
6	0.23	0.03	0.22	0.02
7	1.45	0.15	1.31	0.09
8	10.76	0.82	9.27	0.46
9	95.01	5.29	76.21	2.76
10	673.17	24.51	520.12	12.51

The time taken to compute the Chang and Marr index using a naïve implementation for the Hamming and edit distance can be seen under the columns titled Naïve. That which is computed using the libFLASM library can be seen under the columns titled libFLASM. It is evident that using libFLASM significantly decreases the time taken to compute the Chang and Marr index. This can clearly be seen as the value of *q* increases, when we obtain an implementation which is faster by more than an order of magnitude.

## Conclusions

FLASM is a generalisation of the classic ASM problem and, hence, has numerous direct applications in computational molecular biology and elsewhere. In this article, we presented libFLASM, a free open-source C++ library aimed at solving the FLASM problem under the edit and Hamming distance models. Specifically, given a pattern *x* of length *m*, a text *t* of length *n*≥*m*, an integer *ℓ*≤*m*, and an integer *k*<*ℓ*, it finds all factors of *t* that are at a distance at most *k* from any factor of length *ℓ* of *x*. The main advantage of libFLASM is that it implements state-of-the-art algorithms to achieve a time complexity of $\mathcal {O}\left (m\lceil \frac {\ell }{w}\rceil n\right)$, and a space complexity of $\mathcal {O}\left (m\lceil \frac {\ell }{w}\rceil \right)$, which are independent of the distance threshold *k* and the alphabet size.

libFLASM is freely distributed and can be incorporated easily into any computational pipeline. As proof of concept, we incorporated libFLASM into BEAR, a state-of-the-art tool to improve the accuracy of MCSA in terms of the inferred likelihood-based phylogenies. Furthermore, we incorporated libFLASM into MoTeX-II, a state-of-the-art tool for ME of patterns longer than was previously possible. Finally, we showed how libFLASM can be used efficiently for ACSM with high error ratios as well as implementing the Chang and Marr index. The extensive experimental results presented here suggest that other applications could benefit from using libFLASM, and thus further maintenance and development of libFLASM is desirable.

## Availability and requirements

- **Project name:** libFLASM
- **Project home page:** https://github.com/webmasterar/libFLASM
- **Operating system:** GNU/Linux
- **Programming language:** C++
- **Other requirements:** N/A
- **License:** GNU GPL

## Abbreviations

ACSM: Approximate circular string matching
ASM: Approximate string matching
FLASM: Fixed-length approximate string matching
MCSA: Multiple circular sequence alignment
ME: Motif extraction
MSA: Multiple sequence alignment
